# Mental Fatigue and Spatial References Impair Soccer Players' Physical and Tactical Performances

**DOI:** 10.3389/fpsyg.2017.01645

**Published:** 2017-09-21

**Authors:** Diogo Coutinho, Bruno Gonçalves, Bruno Travassos, Del P. Wong, Aaron J. Coutts, Jaime E Sampaio

**Affiliations:** ^1^Sport Sciences, Exercise and Health Department, University of Trás-os-Montes and Alto Douro Vila Real, Portugal; ^2^Department of Sports Sciences, Universidade da Beira Interior Covilhã, Portugal; ^3^Sport Science Research Center, Shandong Sport University Jinan, China; ^4^Human Performance Research Centre, University of Technology Sydney Ultimo, NSW, Australia

**Keywords:** team sports, GPS, spatial references, time-motion, task constraints

## Abstract

This study examined the effects of mental fatigue and additional corridor and pitch sector lines on players' physical and tactical performances during soccer small-sided games. Twelve youth players performed four Gk+6vs6+Gk small-sided games. Prior to the game, one team performed a motor coordination task to induce mental fatigue, while the other one performed a control task. A repeated measures design allowed to compare players' performances across four conditions: (a) with mental fatigue against opponents without mental fatigue in a normal pitch (MEN), (b) with mental fatigue on a pitch with additional reference lines (#MEN); (c) without mental fatigue against mentally fatigued opponents on a normal pitch (CTR); and (d) without mental fatigue on a pitch with reference lines (#CTR). Player's physical performance was assessed by the distance covered per minute and the number of accelerations and decelerations (0.5–3.0 m/s^2^; > −3.0 m/s^2^). Positional data was used to determine individual (spatial exploration index, time synchronized in longitudinal and lateral directions) and team-related variables (length, width, speed of dispersion and contraction). Unclear effects were found for the physical activity measures in most of the conditions. There was a small decrease in time spent laterally synchronized and a moderate decrease in the contraction speed when MEN compared to the CTR. Also, there was a small decrease in the time spent longitudinally synchronized during the #MEN condition compared to MEN. The results showed that mental fatigue affects the ability to use environmental information and players' positioning, while the additional reference lines may have enhanced the use of less relevant information to guide their actions during the #MEN condition. Overall, coaches could manipulate the mental fatigue and reference lines to induce variability and adaptation in young soccer players' behavior.

## Introduction

Small-sided games (SSGs) have been used as a training method for developing physical, technical and tactical skills in soccer players (Casamichana and Castellano, 2010; Travassos et al., 2014; Gonçalves et al., 2016b). One reason for this popularity is that when task constraints are manipulated appropriately in the design of SSGs it is possible to concurrently train collective behaviors whilst also providing a specific physical stimulus (Gonçalves et al., 2016a). Accordingly, the pitch size and number of players were identified as key constraints to understand how players adapt their behavior (Folgado et al., 2014b; Gonçalves et al., 2016b). For example, increasing the number of players from 2-a-side to 5-a-side seems to increase movement regularity (Aguiar et al., 2015). More recently there are studies showing that players may adapt their movement behaviors according to manipulations on pitch spatial constraints. For example, Gonçalves et al. (2016a) showed that players' physical and tactical performances were altered by modifying the available pitch areas during SSGs (i.e., restricted-space: allowing players to only move within one pitch zone; contiguous-space: allowing players to move to one neighbor zone; and, free-space: unrestricted movement through all zones). The free-spacing promoted greater collective behavior such as increased player movement synchronization and regularity, and also greater physical and physiological responses compared to the restricted and contiguous space conditions (Gonçalves et al., 2016a).

On the basis of these findings, coaches can restrict the space available to players to stabilize the distances between players in different team sub-units with lower effort, or in contrast increase the free space to improve team synchronization with higher physical effort (Gonçalves et al., 2016a). Using this logic, coaches frequently provide pitch reference lines during SSGs to control the space available to players and guide their positioning (Williams, 2013). An example of this spatial constraint may be to divide the pitch into corridors and sectors, with the aim of increasing team movement synchronization and the players' awareness of their positional role. The intent of this approach is to facilitate players to adopt more collective behaviors, however, to our knowledge no study has yet empirically assessed this issue.

It has recently been shown that soccer performance is negatively affected by mental fatigue by reducing running performance at lower speeds (Smith et al., 2015; Van Cutsem et al., 2017), impairing soccer-specific physical and technical performances during SSGs and technical-based drills (Badin et al., 2016; Smith et al., 2016a) and poorer accuracy and speed of decision-making during film-based soccer simulations (Smith et al., 2016b). In general, these studies showed that mental fatigue affects players' ability to interact with the environmental information (Smith et al., 2016b), with possible impairment in the attentional focus (Boksem et al., 2005; Smith et al., 2016a) and physical performance (Badin et al., 2016). Whilst the available studies have provided new insights into the effect of mental fatigue on soccer performance, the methods used to induce mental fatigue have had low ecological validity (i.e., a computerized Stroop-Color Name task of 30 to 90 min was used). Therefore, to overcome these limitations and to examine the translation of these laboratory studies to the field, more ecologically valid methods of inducing mental fatigue are required (Smith et al., 2016b).

At present, no studies have yet examined the effect of mental fatigue on players' tactical behavior. Previous studies have shown SSGs to be a useful tool for assessing the players' tactical behavior during ecological situations, such as the inter-team level of coordination or team dispersion (Folgado et al., 2014a; Gonçalves et al., 2016b), as allowing researchers to better understand how players adapt their movement behavior to different informational constraints. Furthermore, recent research has shown that soccer players may impair their abilities to identify and use the environmental information when mentally fatigued (Smith et al., 2016b) and, therefore, there is a need to identify strategies that may attenuate or help players to guide their movement behavior when mentally fatigued (Smith et al., 2016b). In this sense, pitch spatial references might help to guide players with mental fatigue, however, there is lack of information regarding how pitch spatial reference lines might affect player behaviors during SSGs. Moreover, previous studies have used protocols that poorly represent the ecological setting. To address these issues, this study aimed to identify the effects of mental fatigue and additional corridor and sectorial pitch lines on players' physical and tactical performances during soccer SSGs. Accordingly, we have hypothesized that an ecological task induces mental fatigue based on its higher cognitive demands, such as sustained attention, cognitive processing and perceptual skills. Knowing that mental fatigue is likely to modify players' abilities to use and interact with the environmental information (Boksem et al., 2005). Thus, we also hypothesized that mental fatigue impairs the time that players spent synchronized. At the end, we also assume that additional pitch corridor and sectorial lines modify players' movement behaviors when mentally fatigued. With the decrease ability to use environmental information, the additional spatial references increase the number of information available, therefore, it was hypothesized that players' positional decisions, such as team dispersion and areas explored, are impaired by the increased quantity of information.

## Methods

### Participants

Twelve highly trained amateur youth soccer players (age = 15.9 ± 0.8 y; height = 172.8 ± 5.2 cm; body mass = 59.5 ± 5.2 kg; maximum heart rate = 185.8 ± 10.9 bpm; peak running speed = 23.3 ± 2.8 km.h^−1^; years of experience = 8.9 ± 2.4 year) participated in this study. All players were members of the same team, performed 3 training sessions (90 to 105 min/session,) and played an official eleven-a-side game during the weekend at a regional playing standard in a regular football field (104 × 64 m) (Castellano et al., 2016). Players had trained in the club for 5.0 ± 2.6 years and have 42 weeks of training a year (Fenner et al., 2016). Two goalkeepers participated in each SSG but were not included in the analysis. An informed and written consent was provided to the coaches, players, and their parents, as well as by the club, before the beginning of the study. All participants were notified that they could withdraw from the study at any time. The study protocol followed the guidelines and was approved by the Ethics Committee of the Research Centre for Sport Sciences, Health and Human Development, based at Vila Real (Portugal) and conformed to the recommendations of the Declaration of Helsinki. The sample size was calculated with G^*^Power (Version 3.1.5.1 Institut für Experimentelle Psychologie, Düsseldorf, Germany) for an effect size of 1.05, an α of 0.05, and a power of 0.8 (1–β) (Faul et al., 2007; Ric et al., 2016a). The total sample size computed by this method was 24, that consists in a minimum of 12 subjects in each group.

### Study design

**Figure 1:**
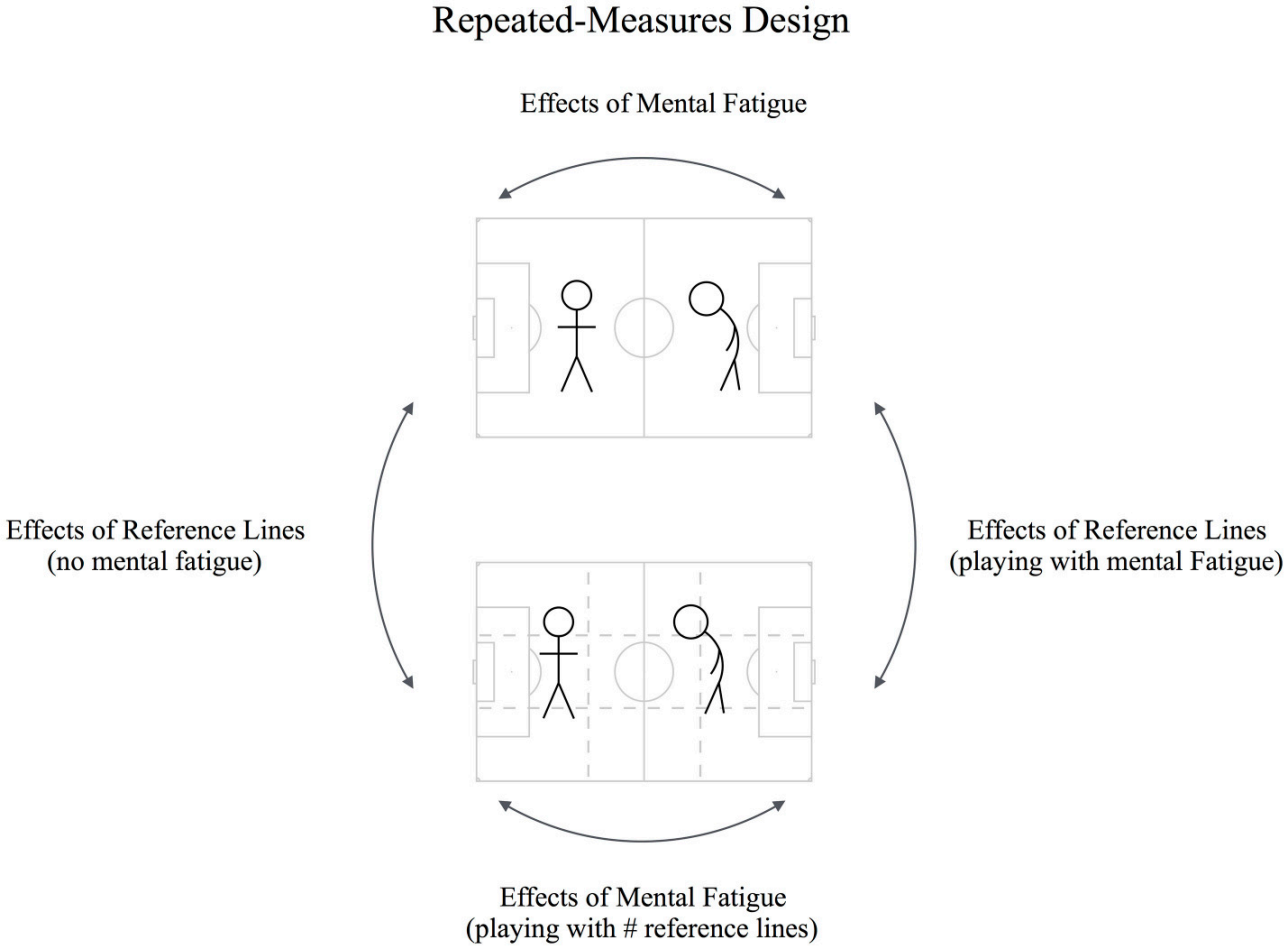
Repeated measures design for the SSG scenarios.

This study used a convenience sample and a counter-balanced crossover design (Badin et al., 2016), using mental fatigue and pitch reference lines as individual and game constraints (see Figure 1), respectively. Each soccer player performed four Gk+6vs6+Gk SSGs. This format was used on the basis that it provides the main team structure for the 11-a-side (1 goalkeeper, 2 defenders, 3 midfielders and 1 forward) and it is widely used during the youth players development (Barbero-Alvarez et al., 2017). Also, previous research has shown that team's tactical performance is only relevant with, at least, a SSG format with 4-a-side (Aguiar et al., 2015). Prior to the SSG, one team performed a motor coordination task to induce mental fatigue, while the other team performed a control task (Badin et al., 2016). The repeated measures design allowed to compare performances across four conditions: (a) with mental fatigue against opponents without mental fatigue in a normal pitch (MEN), (b) with mental fatigue on a pitch with additional reference lines (#MEN); (c) without mental fatigue against mentally fatigued opponents on a normal pitch (CTR); and (d) without mental fatigue on a pitch with reference lines (#CTR). Players' psychological states at the beginning and the final of both the control and mental tasks were measured using the CR10-Scale ratings of perceived exertion (RPE) to identify the players' effort in each task, and with the visual analog scale to understand the level of mental fatigue. Moreover, to inspect the effects of both tasks on the neuromuscular performance, the countermovement jump was assessed with a portable optical timing system (Optojump, Microgate, Bolzano, Italy).

### Procedures

**Figure 2:**
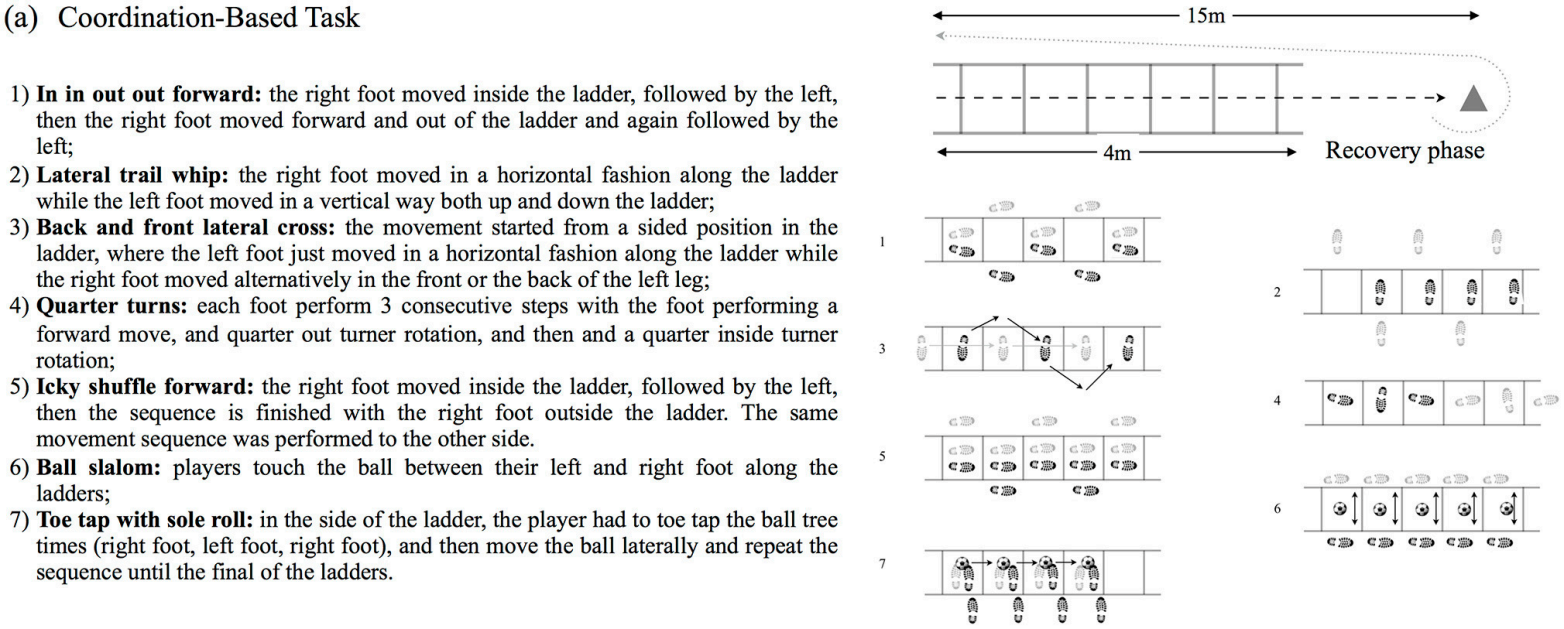
Representation of mental fatigue task (coordination-based task).

**Figure 3:**
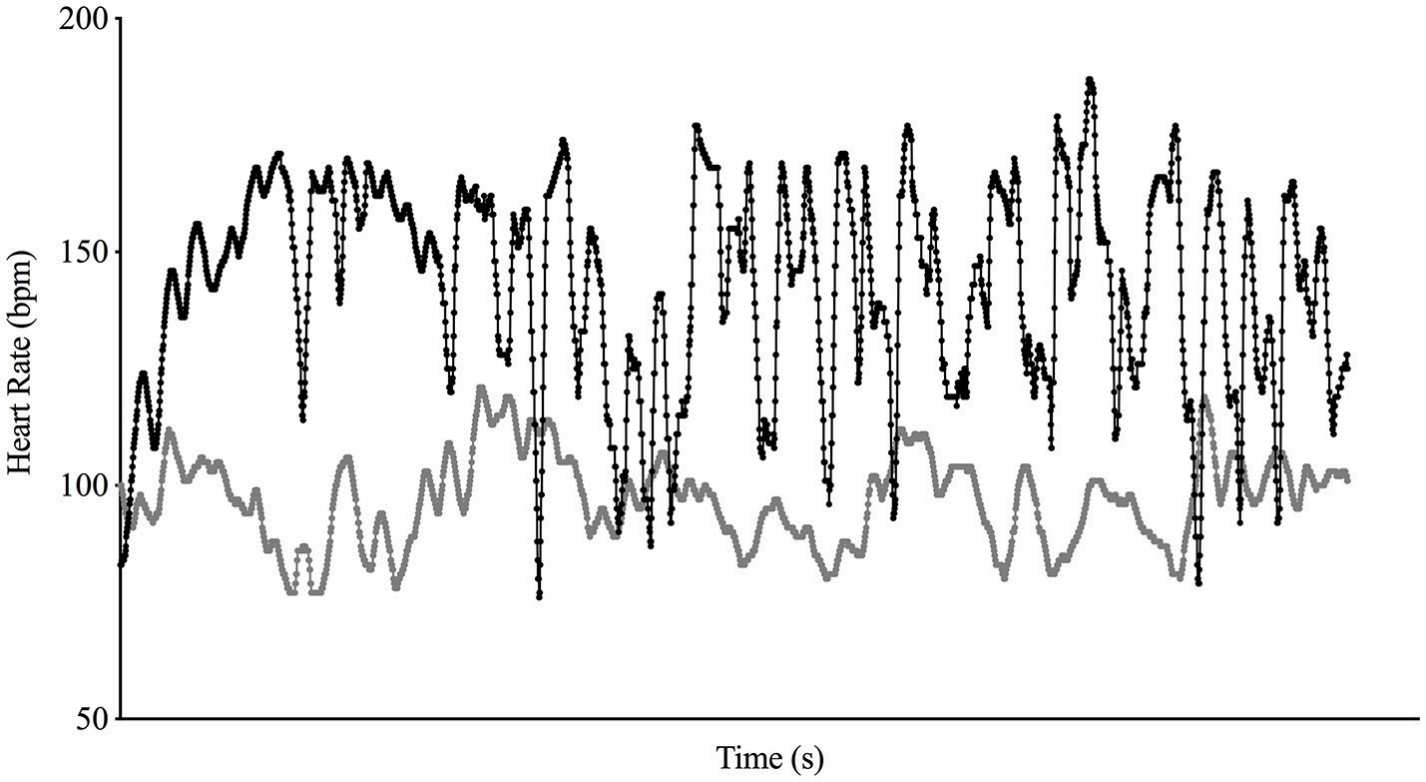
Representation of the heart rate responses to the task used to induce mental fatigue (black line) and the control task (gray line) for the same player.

The testing procedures were performed across five sessions, one per day and interspersed by two resting days. The first session was used for familiarization purposes and the following sessions were used to perform the SSG conditions in a randomized order. In the first session, players were familiarized with the control and mental fatiguing tasks. The task required 20-min of whole-body coordination task (Demirakca et al., 2016; Henz and Schollhorn, 2016), requiring motor coordination, sustained attention, cognitive processing and perceptual skills. This approach systematically stimulated the brain to adapt to unfamiliar challenges using different combinations of motor and cognitive activities (Demirakca et al., 2016). Each participant was required to perform seven different exercises in a ladder drill (see Figure 2). To increase the task attentional and cognitive demands, players were also required to perform these movements while juggling a tennis ball. As soon as the participant's performance increased, a new exercise was introduced. For the control condition, players were required to perform light general aerobic exercises such as skipping, jogging, running backwards, and side stepping. These drills were selected due to their low cognitive demand and similar physical pattern as the mental fatigue task. Figure 3 shows a typical example of heart rate responses from the same player to the mental fatigue and to the control task. In each testing session, one team performed the mental fatigue task and the other team performed the control task of the same duration.

The head coach selected the twelve best players from the team according to their technical, tactical and physical skills and then divided them into two balanced teams taking into account their playing positions (Casamichana and Castellano, 2010). Prior to each SSG, there was a standardized 15-min warm-up based on running and a ball possession game (6-a-side without goals). The games were played on an official artificial turf 62 × 43 m pitch with official 7-a-side goals (Travassos et al., 2014). For the SSG using reference pitch lines, the original pitch was divided using two vertical and two horizontal lines that split the pitch into nine equal sections of 21.3 × 14.3 m. All SSGs lasted for a total of 24-min including 3 x 6 min bouts interspersed with a 3-min passive recovery. Players performed each SSG according their own playing positional role as determined by the head coach. Several balls were placed around the field to ensure its replacement as fast as possible and no coach feedback or encouragement was allowed. All sessions started at the same time of the day and were completed within the same duration (90 min). The repeated measures design from this study required that each subjects' responses were measured in 12 different SSG bouts.

### Data collection

#### Psychological states

The CR10-scale (RPE) was recorded before and after the control and mental fatigue tasks to identify the requirement of both tasks (control and mental fatigue task), and at the final of the SSGs. Mental fatigue was assessed using the visual analog scale (VAS) immediately prior to, and following each treatment condition, and again in the end of the SSG using a 100-mm VAS. The visual analog scales has been reported as a valid and reliable mean to measure mental fatigue (Lee et al., 1991).

#### Neuromuscular performance

To assess the impact of the mental fatigue and control treatment on neuromuscular performance, a countermovement jump (CMJ) was measured before and after the control and mental fatigue tasks. The CMJ was assessed using a portable optical timing system (Optojump, Microgate, Bolzano, Italy), according to the protocol described by Bosco et al. (1983).

#### Physical activity and positional data

Positional data, accelerations and distance covered during SSG were gathered using 15 Hz global positioning system units (SPI-PRO, GPSports, Canberra, ACT, Australia) (Johnston et al., 2014). The units were placed into appropriate elastic harnesses that placed the device on the upper back of each participant (Coutts and Duffield, 2010). The players' latitude and longitude coordinates obtained through games units were exported and computed using appropriate routines in Matlab® (MathWorks, Inc., Massachusetts, USA). Additionally, missing data were re-sampled and tracking error noise was reduced by smoothing the data based on a 3 Hz Butterworth low pass filter (Folgado et al., 2014a).

The positional data of the players were used to determine the team's width and length (Folgado et al., 2014b), the speed of team contraction and dispersion (Ric et al., 2016b), the spatial exploration index (Gonçalves et al., 2016a), and the time that teammates dyads spent synchronized in both longitudinal and lateral directions. These two last variables were calculated with the relative phase with Hilbert transform (Palut and Zanone, 2005; Folgado et al., 2015) and applied for all possible dyads for the six outfield teammates (i.e. for 6 teammates, there were 15 possible dyads). The near-in-phase synchronization of each dyad was quantified by the percentage of time spent between −30° to 30° bin (near-in-phase mode of coordination) (Folgado et al., 2014a).

The distance covered was computed for each game and then divided by the bout duration in order to obtain the meters covered per minute. In addition, the number of all accelerations were measured according to type (acceleration or deceleration) and category (0.5-3.0 m/s^2^ and > -3.0 m/s^2^) (Dalen et al., 2016; Russell et al., 2016).

#### Statistical analysis

Prior to the comparisons among game conditions, all processed variables were log-transformed to reduce the non-uniformity of error. A descriptive analysis was reported as mean and standard deviations for all the considered variables (the mean shown is the back-transformed mean of the log transform). A one-way repeated measures ANOVA was performed to identify differences in physical and positional variables according to game scenarios comparisons. The sphericity was assessed using the Mauchly's test and, when necessary, the Greenhouse-Geisser correction procedure was used to adjust the degrees of freedom. The differences in team length and team width according to game scenario were assessed through one-way ANOVA. Pairwise differences were assessed with a Bonferroni post-hoc. The CMJ, visual analog scale and RPE changes between pre- and post-test were compared using paired t-tests. The inferential analyses were carried in SPSS Software® and statistical significance was maintained at 5%. The comparisons among game conditions, as well as the comparisons between both tasks were also described using standardized mean differences with 95% confidence intervals (Hopkins et al., 2009; Cumming, 2012, 2013). Differences in group means and between the control and mental fatigue tasks were expressed in percentage units with 95% confidence limits (CL). Smallest worthwhile differences were assessed using the standardized units multiplied by 0.2. Uncertainty in the true effects of the conditions were evaluated through non-clinical magnitude-based inferences. Magnitudes of clear effects were considered as the following scale: >5%, unclear; 25 to 75%, possible; 75 to 95%, likely; 95 to 99%, very likely; >99%, most likely (Hopkins et al., 2009). Thresholds for effect size statistics (Standardized differences in terms of Cohen's *d*) were: <0.2, trivial; 0.6, small; 1.20, moderate; 2.0, large; and >2.0, very large (Hopkins et al., 2009).

## Results

**Figure 4:**
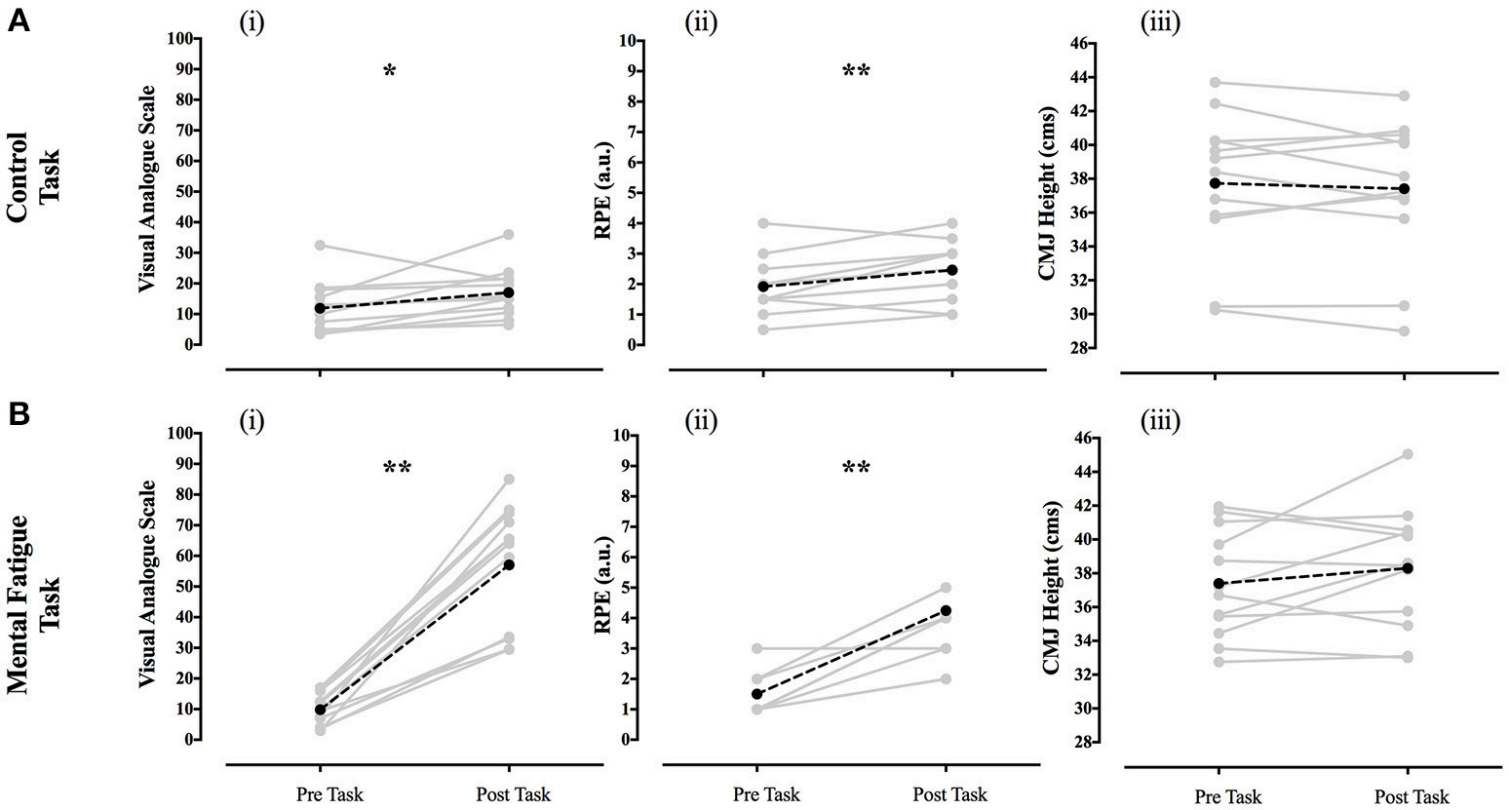
Effects of the control **(A)** and mental fatigue task **(B)** on Visual Analog Scale (i), RPE (ii), and CMJ jumping height (iii) gray solid lines indicated responses of individual participants; black dotted lines indicated mean value. RPE, rate of perceived exertion; CMJ, countermovement jump. ^*^p < 0.05; ^**^p < 0.01.

The effects of the control and mental fatigue tasks (treatment conditions) on VAS, RPE and CMJ performance are shown in Figure 4. Overall, higher values were found in the perception of mental fatigue following the mental task as compared with the control task. Accordingly, after the mental fatigue task, there was a most likely increase (537%) in perceptions of mental fatigue (very large effects: Cohen's d; ± 95% confidence limits: 3.2; ± 0.5; *p* = 0.001) and a most likely ~155% increase in RPE (very large effect, 2.3; ± 0.5; *p* = 0.001). In contrast, the control task elicited a very likely increase (~57%) in perceptions of mental fatigue (moderate effect, 0.6; ± 0.3; *p* = 0.025) and a likely positive ~24% increase in RPE values (small effect, 0.5; ± 0.4; *p* = 0.006).

### Effects of mental fatigue task

**Figure 5:**
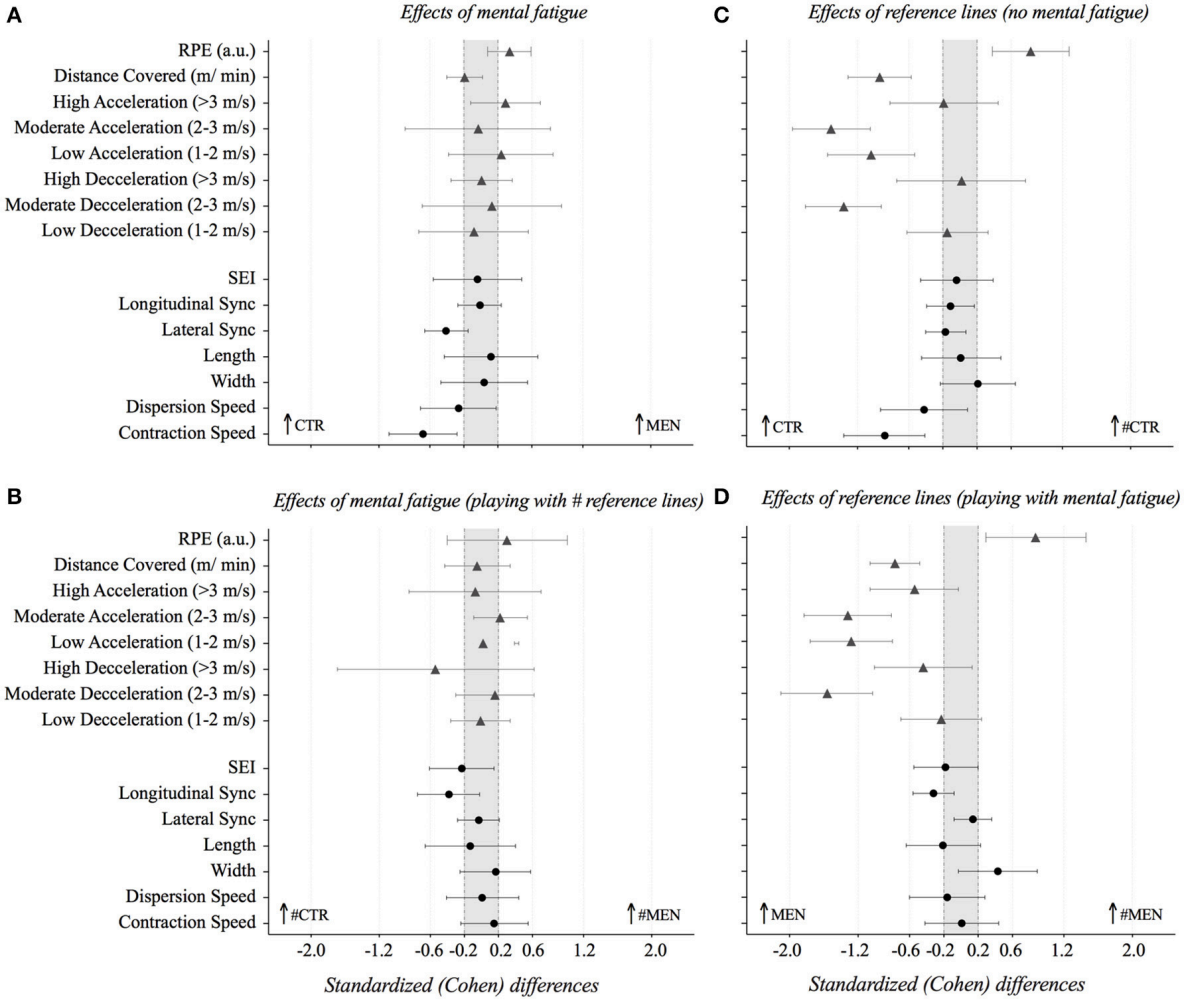
Standardized (Cohen's *d*) differences in physical and positional variables according to mental and pitch reference lines effects: **(A)** effects of mental fatigue (CTR compared to MEN); **(B)** effects of mental fatigue playing with # reference lines (#CTR compared to #MEN); **(C)** effects of references lines without mental fatigue (CRT compared to #CTR); and **(D)** effects of reference lines with mental fatigue (MEN compared to #MEN). Error bars indicate uncertainty in the true mean changes with 95% confidence intervals. S, Small effects; M, Moderate effects; L, Large effects; VL, Very Large effects.

Apart from RPE, mostly unclear effects were observed between both conditions on physical performance measures (see Table 1 and Figure 5A). In this sense, MEN showed a likely increase in the RPE (small effects, 0.3; ±0.3; *p* = 0.012) as compared with the CTR. With the tactical-related variables, the CTR condition elicited a possible increase in the time that dyads spent synchronized in the lateral direction (~20%, small effect, 0.4; ±0.3; *p* = 0.001), a possible increase (~6%) and a very likely increase (~14%) in the contraction speed (moderate effect, 0.7; ±0.4; *p* = 0.004) compared to the MEN condition. The results have also revealed a possible increase in the speed of dispersion in the CTR in relation to the MEN condition.

**Table 1:** Descriptive and statistical analysis for physical and positional variables according to SSG conditions.

**Variables**		**Game Conditions**				**Difference in means (%; ±95% CL) Uncertainty in the true differences**			
		**CTR**	**MEN**	**#CTR**	**#MEN**	**Effects of mental fatigue (CTR-MEN)**	**Effects of mental fatigue with # reference lines (#CTR-#MEN)**	**Effects of reference lines with no mental fatigue (CTR-#CTR)**	**Effects of reference lines with mental fatigue (MEN-#MEN)**
**Physical Variables**	RPE (a.u.)	7.00 ± 1.21	7.58 ± 1.31	8.50 ± 1.62	8.92 ± 1.08	8.2; ± 6.4 (↑ L) *p* = 0.012	6.1; ± 14.7 (U)	21.2; ± 12.6 (↑ML) *p* = 0.001	−18.9; ± 13.8(↑VL) *p* = 0.003
	Distance Covered (m/m)	115.01 ± 13.77	112.19 ± 13.60	101.54 ± 14.64	101.02 ± 15.47	−2.5; ± 3.3 (↓P)	−0.6; ± 5.3 (U)	−12.0; ± 4.4 (↓ML) *p* = 0.001	−10.3; ± 3.7 (↓ML) *p* = 0.001
	High Acc. (> 3 m/s^2^)	2.17 ± 1.52	2.61 ± 1.54	1.56 ± 1.23	1.44 ± 1.31	18.1; ± 33.5 (U)	−3.6; ± 38.6 (U)	−9.7; ± 32.1 (U)	−26.7; ± 22.0 (↓ L) *p* = 0.001
	Moderate Acc. (2−3 m/s^2^)	22.67 ± 13.96	21.94 ± 12.32	7.47 ± 2.97	8.22 ± 3.06	−1.9; ± 48.2 (U)	12.1; ± 18.3 (↑P)	−61.9; ± 11.2 (↓ML) *p* = 0.001	−56.5; ± 14.1 (↓ML) *p* = 0.001
	Low Acc. (1−2m/s^2^)	28.03 ± 7.01	29.42 ± 6.63	20.67 ± 5.86	20.86 ± 6.25	5.9; ± 15.6 (U)	0.7; ± 11.1 (U)	−27.1; ± 11.3 (↓ML) *p* = 0.001	−30.7; ± 9.5 (↓ML) *p* = 0.001
	High Dec. (> 3 m/s^2^)	4.56 ± 2.45	4.58 ± 2.68	3.81 ± 1.89	3.33 ± 1.97	0.9; ± 22.5 (U)	−25.5; ± 50.1 (U)	0.9; ± 41.1 (U)	−27.6; ± 31.2 (↓ L) *p* = 0.004
	Moderate Dec. (2−3 m/s^2^)	34.11 ± 23.08	34.58 ± 21.06	10.61 ± 4.13	10.61 ± 3.98	8.5; ± 58.1 (U)	7.7; ± 23.0 (U)	−62.0; ± 12.2 (↓ML) *p* = 0.001	−62.3; ± 12.7 (↓ML) *p* = 0.001
	Low Dec. (1−2 m/s^2^)	29.47 ± 8.00	29.14 ± 9.38	27.83 ± 7.30	27.5 ± 7.13	−2.1; ± 16.1 (U)	−0.3; ± 10.0 (U)	−4.4; ± 14.0 (U)	−5.5; ± 15.6 (U)
**Positional Variables**	Space Exploration Index (m)	11.80 ± 2.02	11.79 ± 2.48	11.75 ± 2.18	11.35 ± 2.18	−0.7; ± 8.3 (U)	−3.6; ± 5.9 (↓P)	−0.6; ± 7.5 (U)	−3.6; ± 7.4 (U)
	Longitudinal Sync (%)	66.42 ± 1.11	66.46 ± 13.47	64.60 ± 9.86	62.00 ± 14.66	−0.3; ± 5.6 (U)	−5.8; ± 5.4 (↓L)	−2.0; ± 5.1 (↓P)	−7.5; ± 5.3 (↓ L) *p* = 0.009
	Lateral Sync (%)	37.91 ± 12.91	32.72 ± 10.31	35.31 ± 12.26	34.51 ± 10.92	−13.4; ± 7.9 (↓P) *p* = 0.001	−1.4; ± 9.8 (U)	−6.3; ± 8.5 (↓P)	5.5; ± 8.7 (↑P)
	Length (m)	18.90 ± 3.08	19.36 ± 3.71	18.98 ± 3.34	18.62 ± 3.67	2.1; ± 9.4 (U)	−2.3; ± 9.2 (U)	0.3; ± 8.4 (U)	−4.0; ± 8.2 (U)
	Width (m)	19.21 ± 2.95	19.28 ± 2.49	19.93 ± 3.23	20.31 ± 2.37	0.6; ± 7.3 (U)	2.6; ± 6.8 (U)	3.5; ± 7.4 (U)	5.6; ± 6.1 (↑ L)
	Dispersion Speed (m/s)	0.34 ± 0.07	0.32 ± 0.06	0.32 ± 0.08	0.32 ± 0.07	−5.6; ± 9.3 (↓P)	0.3; ± 10.8 (U)	−9.2; ± 10.7 (↓ L)	−3.5; ± 9.3 (U)
	Contraction Speed (m/s)	0.32 ± 0.07	0.32 ± 0.07	0.27 ± 0.05	0.28 ± 0.05	−14.2; ± 10.2 (↓VL) *p* = 0.004	3.5; ± 9.2 (U)	−16.8; ± 8.2 (↓ML) *p* = 0.001	0.3; ± 8.8 (U)

*CL, confidence limits; ↑, increase; ↓, decrease; U, Unclear; T, Trivial; P, Possible; L, Likely; VL, Very Likely; ML, Most Likely*.

### Effects of mental fatigue (playing with # reference lines)

Overall the results revealed non-significant differences in the studied variables, however some trends could be identified (Table 1 and Figure 5B). In this sense, there was a possible ~12% increase in the number of moderate accelerations with #MEN compared to the #CTR condition. When playing with #MEN, it was also found a possible ~4% decrease in the spatial exploration index (small effect, 0.2; ± 0.4) and likely ~6% decrease the time spent synchronized in the longitudinal direction (small effect, 0.4; ± 0.4) compared with the #CTR condition.

### Effects of reference lines (without mental fatigue)

Adding the reference lines in the SSG pitch decreased the players' physical performance but increased the players perception of effort (see Table 1 and Figure 5C). Specifically, adding lines to the pitch without mental fatigue, most likely increased (~21%) the RPE (moderate effects, 0.8; ±0.5; *p* = 0.001). However, it was also found that a most likely decrease (~12%) in the mean speed (m/min, moderate effect; *p* = 0.001), a most likely decrease in the number of moderate accelerations, in the number of low accelerations and in the number of moderate decelerations (~27 to 62%; ±9; large, moderate and large effect, respectively; *p* = 0.001) comparing the CTR with the performance in the pitch without reference lines. Additionally, the #CTR condition elicited a most likely decrease (~17%) in the contraction speed (moderate effect, 0.9; ±0.5; *p* = 0.001) compared to CTR. Also, the results shown a likely decrease (~9%) in the dispersion speed when compared the #CTR with CTR conditions.

### Effects of the reference lines (with mental fatigue)

In general, apart from RPE, higher mean values were found in the absence of reference lines on the pitch (see: Table 1 and Figure 5D). Specifically, while there was a very likely increase in the RPE in the #MEN (moderate effects, 0.9; ±0.6; *p* = 0.003) compared to MEN, in turn it most likely decreased the mean speed (m/min), the number of moderate accelerations, the number of low accelerations and the number of moderate decelerations (~10 to 62%; ±8%; moderate, large, large and large effects, respectively *p* = 0.001) compared to MEN condition. Additionally, when mentally fatigued, adding reference lines likely decreased (~8%) the time the players spent synchronized in the longitudinal directions (small effect, 0.3; ±0.2 *p* = 0.009) compared the SSGs performed without lines.

## Discussion

This study examined the effects of mental fatigue and additional corridor and sectorial pitch lines on players' physical and tactical performances during soccer SSGs. Interestingly, independent of the reference lines, the mental fatigue induced similar physical activity profile compared with the SSG performed without mental fatigue. In contrast, the players' tactical behavior was affected with MEN with a decrease in the level of dyadic synchronization (mainly in lateral directions) compared to the CTR. Adding pitch spatial references decreased the physical demands with and without mental fatigue. From the tactical perspective, the reference lines impaired players' movement synchronization in longitudinal displacements when mentally fatigued. An additional novel finding from this study was that the coordination-based task - which is more practically relevant to soccer players – induced the players' perception of mental fatigue to similar levels previously reported in response to the Stroop task (Badin et al., 2016). Therefore, this result confirms our first hypothesis showing that this ecological task can be used by coaches to induce mental fatigue, and concomitantly prepare their players to train under mental fatigue effects.

### Effects of mental fatigue

Previous reports shown that mentally fatigued players perceived the same physical task as being more effortful (Badin et al., 2016), which may lead them to decrease their physical effort in order to be able to perform the task until the end (Smith et al., 2015). However, the comparison of the physical performance measures with and without mental fatigue revealed unclear effects in most variables. These results are in contrast to several recent studies that reported a decrease in the physical performance with mental fatigue (Smith et al., 2015, 2016a; Van Cutsem et al., 2017). Nevertheless, the nature of the physical performance tasks in these studies were different, with increased physical performance being a primary outcome measure in previous research (i.e. increased distances traveled), whilst soccer performance (i.e. players compete with the opponents to maintain the ball possession and create goal-scoring opportunities) being the primary goal in the present study. Considering that mental fatigue negatively affects the players' cognitive attributes (Boksem et al., 2005; Van Cutsem et al., 2017), it is likely that these effects are more evident from the tactical perspective in game-based situations, where players have to interact with the environmental information to unfolding functional movement behaviors (Travassos et al., 2012).

The present results revealed differences in the time spent synchronized in the MEN compared to the CTR condition, confirming our second hypothesis. While no differences were observed for in the longitudinal synchronization, there was a decrease in the time spent synchronized in the lateral synchronization with MEN, which suggests an altered positioning strategy. Other studies have also observed altered positioning characteristics with modification in SSG contextual factors (Folgado et al., 2014a, 2015). For example, Folgado et al. (2014a) previously demonstrated that soccer players are usually more synchronized in the longitudinal direction as result of the goal locations and pitch length. A possible explanation for the changes observed with MEN in this study is that players pay less attention to maintaining lateral synchronization as greater focus is required to maintain high levels of synchronization in the goal-to-goal direction.

### Effects of mental fatigue (playing with # reference lines)

The present results revealed higher mean values in number of moderate accelerations during the #MEN condition compared to the #CTR. Additionally, there was a greater number of high accelerations with MEN compared to the CTR when the SSG was played without reference lines. These observations show that despite being mentally fatigued, players are still able to complete moderate to high intensity actions when required. These results agree with recent observations that demonstrated soccer players to down-regulate the speed of lower intensity activities (Smith et al., 2015), in order to have resources available that allow them to effectively respond to high-effort tasks.

In both training and match play, soccer players continuously interact with the environment to make decisions, quite often whilst under pressure from the opponents (Nedelec et al., 2012). Due to the high levels of vigilance required for these tasks, player's must maintain high concentration levels to pick up the relevant information from the environment to guide their behavior (Travassos et al., 2012; Smith et al., 2016b). However, most studies have shown that mental fatigue is likely to affect the players cognitive and perceptual skills, such as a reduced ability to gather and use available information (Kato et al., 2009; Guo et al., 2016). Accordingly, it is likely that the players' behavior might become affected when mentally fatigued. Although the results from this study showed similar movement behavior profiles between both conditions, it was possible to identify a higher player spacing exploration index values and time spent synchronized in the longitudinal direction during the #CTR condition compared to the #MEN. These observations suggest that mental fatigue may affect the way of how players interact with the environment. The current findings also suggest that the addition of the pitch reference lines might have amplified these effects. That is, since the pitch reference lines increase the available information to the players, greater demands are placed upon their perceptual skills, which may make it more difficult to make fast decisions to act (Vaeyens et al., 2007). This increased cognitive load on the players may have mediated the changes in the interpersonal coordination among teammates.

### Effects of reference lines (no mental fatigue)

Players reported higher RPE values during the #CTR even though there was a decrease in several player physical performance characteristics (i.e. accelerations and decelerations) during the #CTR when compared to the CTR. Previous reports have found that adding perceptual cues to a task seems to modify players' perception of effort (Blanchfield et al., 2014). In this sense, lower values of RPE have been associated with a broader scope of attention (Lind et al., 2009), however, the additional information provided by the reference lines might have narrowed players' attentional focus, and concomitantly increased their perception of effort. Furthermore, the decrease in the physical performance results agree with previous research that reported a decrease in physical and physiological load when the players performed the SSG with pitch constrained-spacing (Gonçalves et al., 2016a) and suggest that the spatial references may help players to better perceive and control the distances between teammates and opponents. Thus, it is also possible that the decrease found in the #CTR may be related with this increased perception of the distances. Based on these findings, we suggest that coaches add reference lines during training sessions when the goal is to highlight specific movement behaviors while controlling the training load.

It has previously been shown that players exhibit different tactical behaviors when exposed to specific spatial references, such as the target location in futsal (Vilar et al., 2014) or the number and location of available targets to score (Travassos et al., 2014). In this study, the altered spatial references elicited different tactical behaviors. For instance, in the #CTR condition, there was a greater decrease in contraction speed compared to the CTR, as well as high mean values in the dispersion speed. These differences in players tactical behavior with the pitch lines suggest that the players collectively adjusted their behavior through using the reference lines as spatial references to control the distances between them (Fajen, 2007). Indeed, it may be that the players on both teams use the reference lines to assist them to better perceive the distance between them during the dispersion/contraction moments which resulted in decreased movement speed.

### Effects of the reference lines (playing with mental fatigue)

The reductions in the speed and accuracy of decision-making previously reported in soccer players (Smith et al., 2016b) are likely related to the impaired ability to identify and use the environmental information (Lorist, 2008). However, this reduced decision-making ability appears to be result of the decreased ability to suppress the irrelevant stimulus with higher levels of mental fatigue (Boksem et al., 2005; Lorist, 2008; Faber et al., 2012). Therefore, it seems plausible to assume that mentally fatigued players may also use inappropriate information to sustain their actions (Boksem et al., 2005; Lorist, 2008; Faber et al., 2012), leading to different self-emerging behaviors.

Whilst the reference lines were added to the pitch to guide the players' behavior, it is possible that these may have increased the irrelevant information available to the mentally fatigued players and therefore exacerbated the effects of mental fatigue. In fact, the observed decreases in physical game-related performance, in time spent longitudinally synchronized, and increased RPE values during the #MEN compared to the MEN suggests that the increased perceptual irrelevant stimulus induced by the combined condition of pitch lines and mental fatigue modified the players' interpretation of the information resulting in altered self-organization patterns between the teammates. Generally, these evidences confirmed our third hypothesis, revealing that adding pitch reference lines may increase the available information, and possibly, increase the use of irrelevant cues by mentally fatigued players.

This study adds novel information regarding the effects of the mental fatigue and spatial reference lines on the players' behavior, however, some limitations of the study should be acknowledged. For example, we used a counter-balanced approach where only one team was under the effect of mental fatigue during each SSG condition, and therefore, it is possible that different results emerged if both teams were under the same effects. Moreover, most of the available researches have focused on analyzing the effects of mental fatigue in SSGs, and different results may emerge during large-sided games and competitive matches (e.g. 9-a-side or 11-a-side). Thus, future studies should analyze the players' behaviors when both teams are mentally fatigued and using different number of players. Furthermore, while this study shows the effects of the reference lines on the players' performance, further studies should also analyze the effects of different task constraints under mental fatigue conditions. Nevertheless, this study showed important implications for soccer training. Accordingly, the task used in this study showed the potential to induce mental fatigue and, therefore, coaches could use it during their training sessions to improve players' responses to mental fatigue. Furthermore, taking into consideration that players ability to use environmental cues under mental fatigue may be impaired, coaches should carefully design training tasks, minimizing the presence of visual cues, once the increased information may enhance the effects of mental fatigue.

## Conclusions

In summary, independently of the pitch reference lines, similar physical game-related activity variables were observed with and without mental fatigue. We also observed that MEN decreased the players' lateral synchronization, possibly as a strategy to maintain high levels of synchronization in the goal-to-goal direction. It also appeared that the MEN influenced the players' ability to perceive and sustain their decisions based on the information from environment, which resulted in different positioning and collective synchrony compared to the CTR condition. The pitch spatial references seem to be a suitable constraint to manage the players' physical effort in the absence of mental fatigue. However, we also observed that when the pitch lines were combined with mental fatigue the players positioning was affected. Finally, the motor coordination task used to induce mental fatigue in this study elicited values that were similar to those reported in studies that used in non-specific computerized tasks. These tasks might be useful as a training intervention for mental fatigue in team sport athletes.

Overall, coaches could use mental fatigue and reference lines to induce variability and adaptation in young soccer players' behavior. Finally, the coordination-based task performed in this study might be used as training intervention for mental fatigue in team sport athletes.

## Ethics statement

An informed consent was provided to the coaches, players, and their parents, as well as by the club, before the beginning of the study. All participants were notified that they could withdraw from the study at any time. The study protocol followed the guidelines stated by the Ethics Committee of the Research Centre for Sport Sciences, Health and Human Development, based at Vila Real (Portugal) and conformed to the recommendations of the Declaration of Helsinki.

## Author contributions

DC was involved in the design of the study, data collection, analysis and interpretation, as well as in the draft of the main document. BG was part of the data design and collection, and was also responsible for developing the statistical analysis and manuscript content revision. DW helped in the study design, data interpretation and reviewed the manuscript content, helping to develop the main findings from the physical performance. BT worked on the design of the study, data collection and analysis, and also helped to translate the main findings to a practical point of view. AC participated during the data interpretation, and also had an active role in the manuscript revision, ensuring that all information was properly connected. JS worked on the study draft, conception and design, data analysis and interpretation and revised it critically. All authors approved the final version and agree to be accountable for all aspects of the work.

### Conflict of interest statement

The authors declare that the research was conducted in the absence of any commercial or financial relationships that could be construed as a potential conflict of interest.
